# Densification of agro-residues for sustainable energy generation: an overview

**DOI:** 10.1186/s40643-021-00427-w

**Published:** 2021-08-14

**Authors:** Segun E. Ibitoye, Tien-Chien Jen, Rasheedat M. Mahamood, Esther T. Akinlabi

**Affiliations:** 1 Department of Mechanical Engineering Science, Faculty of Engineering and the Built Environment, University of Johannesburg, P. O. Box 524, Auckland Park, 2006 South Africa; 2 Department of Mechanical Engineering, Faculty of Engineering and Technology, University of Ilorin, P. M. B. 1515, Ilorin, Nigeria; 3 Department of Materials and Metallurgical Engineering, Faculty of Engineering and Technology, University of Ilorin, P. M. B. 1515, Ilorin, Nigeria; 4 Directorate, Pan African University for Life and Earth Sciences Institute, Ibadan, Nigeria

**Keywords:** Biomass, Briquetting, Densification, Fuel, Feedstock, Pelletizing, Sustainable energy

## Abstract

The global demand for sustainable energy is increasing due to urbanization, industrialization, population, and developmental growth. Transforming the large quantities of biomass resources such as agro-residues/wastes could raise the energy supply and promote energy mix. Residues of biomass instituted in the rural and industrial centers are enormous, and poor management of these residues results in several indescribable environmental threats. The energy potential of these residues can provide job opportunities and income for nations. The generation and utilization of dissimilar biomass as feedstock for energy production via densification could advance the diversity of energy crops. An increase in renewable and clean energy demand will likely increase the request for biomass residues for renewable energy generation via densification. This will reduce the environmental challenges associated with burning and dumping of these residues in an open field. Densification is the process of compacting particles together through the application of pressure to form solid fuels. Marketable densification is usually carried out using conventional pressure-driven processes such as extrusion, screw press, piston type, hydraulic piston press, roller press, and pallet press (ring and flat die). Based on compaction, densification methods can be categorized into high-pressure, medium-pressure, and low-pressure compactions. The common densification processes are briquetting, pelletizing, bailing, and cubing. They manufacture solid fuel with desirable fuel characteristics—physical, mechanical, chemical, thermal, and combustion characteristics. Fuel briquettes and pellets have numerous advantages and applications both in domestic and industrial settings. However, for biomass to be rationally and efficiently utilized as solid fuel, it must be characterized to determine its fuel properties. Herein, an overview of the densification of biomass residues as a source of sustainable energy is presented.

## Introduction

Sustainable energy is the backbone for the social–economic expansion of any country. It plays a significant role in national and intercontinental diplomacy. It is a marketable product for earning national and international income, which can fund governmental advancement and innovation programs (Ajimotokan et al. 2019a). Energy is an input into the manufacture of products and services in industrial, transportation, health, education, and agricultural sectors and a device for politics and security. The desire to provide clean, environmentally friendly, renewable, and sustainable energy had continued to increase as an effort to reduce environmental degradation due to the use of fossil fuels for a long time. This is essential to provide healthy living and a green environment.

A sustainable energy system is a reliable, environmentally friendly, and cost-efficient energy source that effectively uses locally available resources as the primary feedstock or raw materials for its generation (Ojolo et al. 2016; Suberu et al. 2012; Ahmad et al. 2016). They are energy that does not cause environmental degradation as experienced with the use of fossil fuels. It is bendable in relation to new technology, profitability, and governmental solutions. Among the renewable energy sources that display sustainability properties, biomass energy exhibited favorable characteristics, which have to be promising and affordable for the past few decades. This energy source had been broadly exploited, possibly because of its abundance, cost-effectiveness, and native nature (Donepudi 2017). Also, because biomass retains a closed carbon cycle with no net rise in atmospheric carbon dioxide, this is due to the replanting operations of the previous harvest, which utilizes the carbon dioxide emitted by conventional energy sources.

The global demand for sustainable energy is increasing due to an increase in urbanization, industrialization, populace, and developmental growth. Regrettably, the obtainable infrastructures for the supply, especially in the rural areas, are limited. From the global assessment, more than half of the human populace has no access to a sustainable form of energy (Ahmad et al. 2016; Muhammad 2019; Manouchehrinejad and Mani 2018; Meda and Dumonceaux 2018; Tuates et al. 2016a). A larger percentage of this population lives in developing countries and is usually underprivileged. They majorly depend on primitive biomass as the main energy source, which had caused health hazards and several indescribable risks. Studies have shown that there are abundant available resources for renewable energy generation in most of the rural areas (Oyedepo et al. 2019). Despite the availability, there is low access to clean energy by the teeming populace. Transforming the abundance of biomass resources such as agricultural wastes, which are most of the time disposed of by dumping and burning them to produce usable energy, could raise the energy supply by promoting an energy mix. The energy potential of these residues can provide job opportunities and income for nations instead of causing environmental hazards.

Biomass resources can be converted into usable energy via several treatments such as densification. The consumption of densification products has increased from 2 million to 37 million tons from 2000 to 2015 due to the increase in global energy demand. This accounted for about 92% increase in energy consumption (Gauthier 2015). Since 2011 when pellets production and consumption reach an equilibrium, many electric power plants in the United Kingdom have made a complete transition to the utilization of solid biomass fuel as feedstock (IEA 2011). In 2013, the global pellet production was led by the EU (50–12.2-million tons) followed by the US (and Canada (31%), China (9%), Russia (7%), and the rest (4%), all cumulated to about 24.5-million tons. The global pellet consumption followed the order—Europe and UK (23.2-million tons), US and Canada (2.7-million tons), Russia (1 million tons), Asia (0.9 million tons), and the rest with about 0.3-million tons (Solorzano et al. 2017). A similar trend was reported in 2016, with total pellet consumption of ~27.8 million tons (Gauthier, et al. 2017). With the recent trend in global energy transition and governmental policy regarding the use of biomass energy, it is anticipated that the consumption of densification products would continue to increase, and making over 50% of the global renewable energy sources (Solorzano et al. 2017; Gauthier et al. 2017).

The consumption of the products of other biomass treatments (such as gasification, anaerobic digestion, pyrolysis, torrefaction) is also increasing in recent years to meet the EU target of 32% renewable energy by 2030. The number of biogas and bio-methane plants in the EU rose to about 17,783 in 2017, with electricity generation of 65,179 GWh (Biogas trends for this year 2021; Scarlat et al. 2018). Biofuel production is also on the increase, with Europe as the highest consumption in the form of biodiesel. The biofuel industries are still in the developmental stage in Europe, with about 8% increase in consumption from 2016 to 2017 (Achinas et al. 2019). The blend of biodiesel in Europe’s fossil fuel rose to about 6.4% in 2019. Germany is the highest biofuel production after Europe, with about 3000 million liters in 2019 and annual consumption of about 2600 million liters (Europe biodiesel market 2021). A reduction in production and consumption was recorded in 2020 due to the Coronavirus pandemic. However, an improvement in production and consumption is anticipated in the coming years (Renewables 2020).

In 2015, the world’s daily consumption of petroleum was about 92 million barrels, making it the major global energy source. This forms about 33% of the global energy generation, followed by coal (24%) and natural gas (21%). The remaining percentage are from renewable energy (19.1%), and nuclear energy (2.6%) (EIA 2021; Annual Reporting on Renewables 2015). Approximately 50% of the global renewable energy sources are derived from biomass—firewood/biochar (23%), biofuel (22%), biogas (5%). The other 50% are derived from hydroelectric, wind, solar, and geothermal energy with about 26, 18, 4, and 2%, respectively (Ren et al. 2014).

Biomass energy makes about 15% of the global total energy supply, and they are majorly used for heating and cooking, especially in developing nations (Rabiu et al. 2019). It has been forecasted that by 2060, the utilization of biomass for energy generation will increase to about 200 exajoules compared to the level of application in the 1990s (Adeleke et al. 2019). Researches have also shown that by 2050, the percentage of renewable energy supply in the total energy used will increase from 55% to about 75%. Therefore, the European Union is determined and currently working to increase the proportion of biomass in the renewable energy supply by up to about fifty percent (Swiechowski et al. 2019).

At present, it is not practicable to completely substitute conventional fuels with renewable energy supply in a justifiable manner. However, using dissimilar biomass as feedstock could advance the diversity of biomass feedstock and energy crops. It is anticipated that the increase in the percentage of renewable energy supply will give rise to an increase in the request for biomass from agro-residues, which will reduce the environmental challenges associated with their disposal.

Presently, agro-waste is among the common resources in developing countries that could elucidate fuel, energy, and environmental problems. It has limited shortcomings, such as low bulk and energy densities, handling problems, irregular sizes, low fixed carbon, high volatile content, low heating value, low combustion efficiency, etc. (Crawford et al. 2015; Sedlmayer, et al. 2018; Pimchuai et al. 2010). Most of the time, these limitations usually make it difficult for biomass to be used as fuel. However, technologies have been developed to minimize, if not eliminate, these limitations. The technologies suggest an attractive medium to exploit some biomass groups for providing for both rural and urban energy needs through densification. Densification is the process of compacting particles together through the application of pressure to form solid fuel. The densification pressure makes raw biomass particles interlock and sticks together during handling, transportation, combustion. These processes include briquetting, pelletizing, bailing, and cubing (Akogu and Waheed 2019). Biomass densification is needed to reduce or eliminate the problems associated with direct biomass utilization. Densification would reduce the high storage capacity and transportation problem associated with direct biomass utilization. It improves the structural homogeneity, energy density, and heating value of raw biomass. It would reduce over-dependency on wood as fuel. Overall, densification would makes biomass appropriate for use for further conversion processes such as thermal pretreatment processes. When raw biomass is compared with densification product, raw biomass exhibit low thermal efficiency, poor combustion efficiency, high moisture content, low calorific value, low energy density, high emission of smoke and greenhouse gases, non-uniform in size and shape, difficulty to harness and utilized, and they generate dust which pose health risk to people in the surrounding.

Therefore, this manuscript presents an overview of biomass densification as a sustainable energy source for different applications. The article is grouped into eight sections. Section 1 is the “Introduction”. A general overview of densification technology is presented in Sect. “Biomass densification”. Section “Forms of biomass densification” discussed the different forms of densification technologies, while Sect. “Characterisation of feedstock and densification products” talks about the characterization of feedstock and densification products. The advantages, disadvantages, and application of densification are itemized in Sect. “Advantages, disadvantages, and application of densification and its products”, while recent research efforts on biomass densification are presented in Sect. “Recent research efforts”. Section “Drawbacks and proposed possible solutions” identifies the drawbacks associated with biomass densification and proposed solutions. Further research recommendations are items in Sect. “Recommendation for further research”. The manuscript ended by enumerating the summary and conclusions in Sect. “Conclusion”.

## Biomass densification

This section discussed the need for biomass densification. The different feedstocks that can be utilized and procedures for densification processes were discussed. The chemistry behind densification processes—effects of pressure and particle sizes were also highlighted in this section. The forms of densification process were enumerated, while the common forms of densification processes were discussed in detail in the next section.

### Need for biomass densification

Handling an enormous quantity of biomass is energy and labor-intensive, which is one of the major financial factors impeding the use of biomass for sustainable energy and heat generation. Biomass densification is a promising solution to the high storage capacity and transportation problem limiting biomass utilization. It improves structural homogeneity, energy density, and automated feedings in continuous boiler systems (Stelte et al. 2010; Chico-santamarta et al. 2012). Densification products such as pellets/briquettes are preferred to wood chips in heating value and moisture content in many ramifications. These products demand fewer containers to transport the same quantity of energy than raw materials (Poyry 2015).

Biomass densification is a recognized mechanical, technological process that is gaining popularity for over a century. The earliest patented biomass densification procedure was recorded in Chicago in 1880 by William Harold Smith (Stelte 2011). The transformation of biomass to solid fuels of high density is possible to elucidate the problem caused by solid waste and high dependence on wood as fuel in developing nations (Akande and Olorunnisola 2018; Tembe et al. 2014). It is an efficient means of exploiting agricultural wastes for clean energy generation and social–economic development (Ikubanni et al. 2019).

### Densification feedstock and mechanism

Today, the feedstock used for densification is mostly wood residues (such as wood chips, wood shavings, and sawdust), grasses (grain residues or energy crops), and agricultural residues (which include agricultural, industrial wastes, and agro-residues). Most of the time, biomass is appraised using density conversion factors of emergent stock, which is frequently calculated in terms of volume in m^3^. Densification of biomass into solid fuels makes the biomass uniform in size and shape for stress-free handling (Oyelaran and Sanusi 2019; Jiang et al. 2016). This makes it fit for use in thermal conversion processes, for example, gasification, co-firing with coal, combustion, and pyrolysis (Bazargan et al. 2014).

Densification mechanism can be classified into five categories: interfacial and attraction forces, formation of solid bridges, capillary pressure, adhesion and cohesion and, mechanical interlocking (Peng et al. 2015; Mitchual 2014). During densification, natural adhesion forces the particles to make close contact while the mechanical pressure makes the particles interlock. These result to the formation of solid bridges through solidification of the glass transition constituents in the particles due to compression and heating. The mechanical pressure melted or softened the natural binder (lignin) during the process of densification, leading to the formation of interlock and solid bridges between the particles. For the period of compaction, solid bridges are formed through sintering, chemical reactions, hardening of the binder, crystallization of the softened constituents, and solidification of the heated substances (Tumuluru et al. 2011,2010). The applied pressure lowers the melting point of the feedstock particles, making them flow towards each other. This leads to an increase in the contact surface area and shifting the melting point to a fresh balance state. If densification pressure is high, it can result in the crushing of feedstock particles, hence, causing the cell structure to open and uncovering the pectin and protein that function as natural binders, which enhance the strength of densification products (Crawford et al. 2015; Mitchual 2014; Bermudez and Fidalgo 2016). At elevated pressure, outstanding strength properties are attained via improved attraction and Van der Waals forces and, H-bonding which reduced the distance between end-to-end particles (Zhai et al. 2018).

Marketable densification is usually carried out using orthodox pressure-driven processes such as extrusion and piston type (Rabiu et al. 2019; Tilay et al. 2015; Mopoung and Udeye 2017; Nicksy et al. 2014). The most common densification processes are briquetting and pelletizing. They manufacture solid fuel with desirable fuel properties. The detail features of the common biomass densification processes are discussed in Sect. “Forms of biomass densification”.

## Forms of biomass densification

This section discussed the different forms of biomass densification. Merit and demerit of each form of densification are also presented. Some essential factors that influence their operation and output products are discussed.

### Briquetting

Briquetting is one of the orthodox densification processes used to manufacture solid fuels (Karunanithy et al. 2012; Kumar et al. 2017). It involves the mixing of feedstock particles and the application of pressure. It is the process of compacting homogenous or non-homogenous loose combustible materials into a product of higher density for fuel-making purposes (Kumar et al. 2017; Oladeji 2015; Ajobo 2014; Supatata et al. 2017). Biomass of low bulk density is transformed into fuel briquettes with high energy concentration and density via brequetting. It improves physico-mechanical and combustion properties (Ajiboye et al. 2016; Tuates et al. 2016b; Oladeji et al. 2016). The high mechanical pressure makes the feedstock particles sandwiched and stick together, ensuring that no separation exists during storage, combustion, and transportation (Promdee et al. 2017; Thulu et al. 2016). Briquetting can be done with or without a bonding agent or adhesive. The binding agents are added to help hold the feedstock particles together, especially biomass material without plasticity (Zubairu and Gana 2014; Ikelle et al. 2014). It is anticipated that the binding material is burnable. However, a non-burnable binder that is efficient in small quantity may be utilized. Some materials used as binders include clay, starch, magnesia lime, tar, pitch, plaster of Paris, asphalt, sulphite liquor, resin, molasses, and cement (Zubairu and Gana 2014). An optimum proportion of binder/adhesive range of 5–25% is recommended to produce high-quality briquettes (Oladeji and Enweremadu 2012; Espuelas et al. 2020; Ajimotokan et al. 2019b). Briquetting can be done with or without the application of heat. Application of heat most of the time improves the mechanical strength of the end products (Deiana et al. 2004; Alhassan and Olaoye 2015).

To adequately understand the appropriateness of feedstock for briquetting, it is crucial to be acquainted with the physico-chemical and thermal characteristics of feedstock that can influence its properties as fuel. Physical properties include void volume, moisture content, and bulk density, while the chemical characteristics include the proximate and ultimate analyses and calorific value. The operating parameters considered during briquetting include pressure, residence time, and temperature, while the feedstock parameters include moisture content, particle shape size, and external additives (Oladeji 2010). These parameters can be optimized so that briquettes of good quality can be produced. The optimum briquetting temperature and pressure range from 100 to 250 °C and 50–250 MPa, respectively, while the optimum residence time is between 4 and 25 min (Stelte 2011; Ahiduzzaman and Sadrul Islam 2013; Alaru et al. 2011; Chou et al. 2009; Marsh et al. 2007). Successful and effective briquetting required feedstock with moisture content ranges of 5–15% and particle size ranges of 1–10 mm (Mopoung and Udeye 2017; Maia et al. 2014).

Based on compaction, briquetting methods can be categorized into three: high pressure, medium pressure (plus heating), and low pressure (with binder) compactions (Oladeji 2015; Grover and Mishra 1996a). In all these briquetting methods, the solid feedstock is the starting resource, and the feedstock particles can roughly be identified in the end product. High-pressure briquetting enhances adhesion and mechanical interlocking between feedstock particles. It brings about the formation of intermolecular bonding at the particle’s contact areas. Lignin (the natural binding agent in biomass) is softened at elevated pressure and temperature, leading to forming an adsorption layer within the biomass feedstock particles. The externally applied force, such as pressure, increases the contact surface area and brings about molecular forces that enhance the bonding strength between the adhering particles. Different bonds are formed during briquetting. These bonds could occur through attractive forces, van der Waals’ forces, cohesion and adhesion forces, and interlocking forces resulting from applied pressure, heat, and binder.

**Fig. 1:**
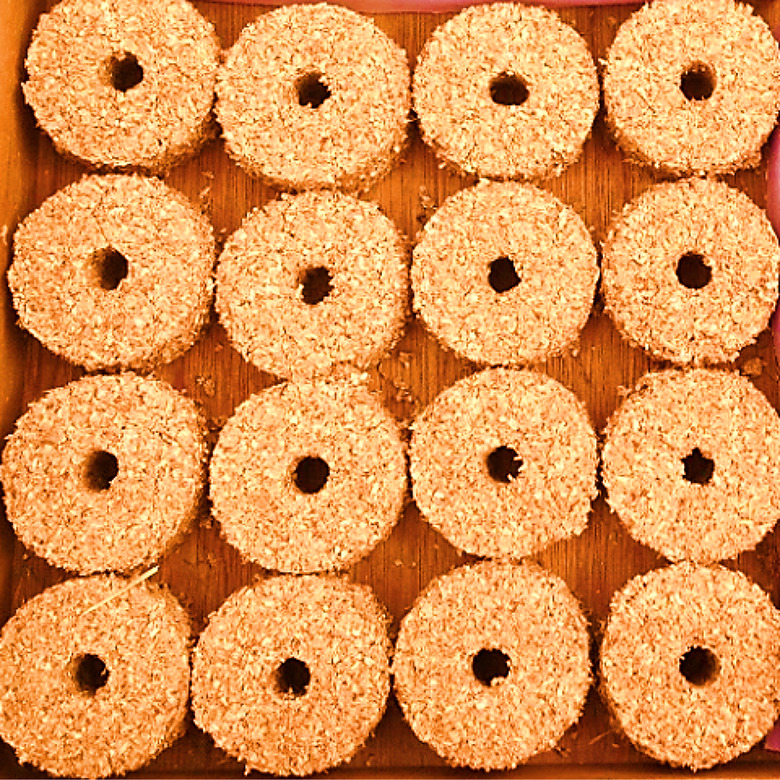
Samples of fuel briquettes (Ajimotokan et al. 2019c)

**Fig. 2:**
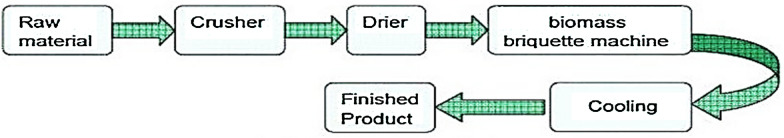
Chart of biomass briquettes manufacture process (Sharma et al. 2015)

The feedstock is compressed in a mold, and the end product of the process is called briquette. Briquette could be made of diverse sizes and shapes depending on the configuration of the mold (Oladeji 2015). Briquette is a solid combustible matter employed as fuel to initiate and sustain a fire (Mohammed and Olugbade 2015). Briquettes fuel is promising because it contained little or no fly ash and sulfur. It has great combustion efficiency, is easy to ignite, and is carefully sized for thorough combustion and long burning time (Alhassan and Olaoye 2015). If manufactured at reduced cost and made available to consumers, it can serve as a substitute for fossil fuel, charcoal, and firewood for home cooking and industrial utilization (Wamukonya and Jenkins 1995; Oyelaran and Tudunwada 2015). Once dried, it can be warehoused at ambient temperature. Storing at elevated temperatures could make briquettes to be too dry and making ignition somehow difficult. However, low storage temperature can soften the briquettes and make them not lasting long during combustion. Figure 1 (Ajimotokan et al. 2019c) shows samples of briquettes, while Fig. 2 (Sharma et al. 2015) shows the chart of biomass briquettes manufacturing process. Briquettes are manufacture using briquetting machine. Piston press and screw press are the two machines that have been repeatedly used to manufacture fuel briquettes. The screw press briquetting method was invented in Japan in 1945 (Grover and Mishra 1996a). Table 1 presents the different types of briquetting machines along with their feature, merit, and demerit.

**Table 1:** Briquetting machine with their features, merits, and demerits

Machine type	Image	Features	Merits	Demerits	References
Piston press		Feedstock is compressed into a die by a to and fro moving ram Extrusion is done by the reciprocating piston Produced briquettes are usually with a concentric hole High-pressure type	Efficient and uniform combustion due to larger surface area Robust with a reputation of long working life They are simply maintained Less wear and tear Power consumption is minimal	Require frequent maintenance Cannot be used to manufacture carbonized briquettes Produced briquettes are not homogeneous	Sharma et al. (2015), Young and Khennas (2003), Ghaffar et al. (2015)
Screw press		Continuously extrude feedstock via an externally heated taper dye Extrusion is done using a specially designed screw Produced briquettes are completely solid Regular, homogeneous, and can withstand greater impact force without crumble High-pressure type	Generate less noise Used for both carbonized and non-carbonized briquettes Produced briquettes of high quality Briquettes are homogeneous and suitable for gasifier	High tear and wear High power consumption Required specific feedstock properties	Tuates et al. (2016a), Grover and Mishra (1996a), Young and Khennas (2003), Ghaffar et al. (2015) With permission
Hydraulic piston press		Driven by an electric motor via a hydraulic system low-pressure type	Light and compacted It can briquettes feedstock with higher moisture content	Slower with lower outputs Usually have a smaller bulk density	Grover and Mishra (1996a), Young and Khennas (2003), Shuma and Madyira (2019)

### Pelletizing

**Fig. 3:**
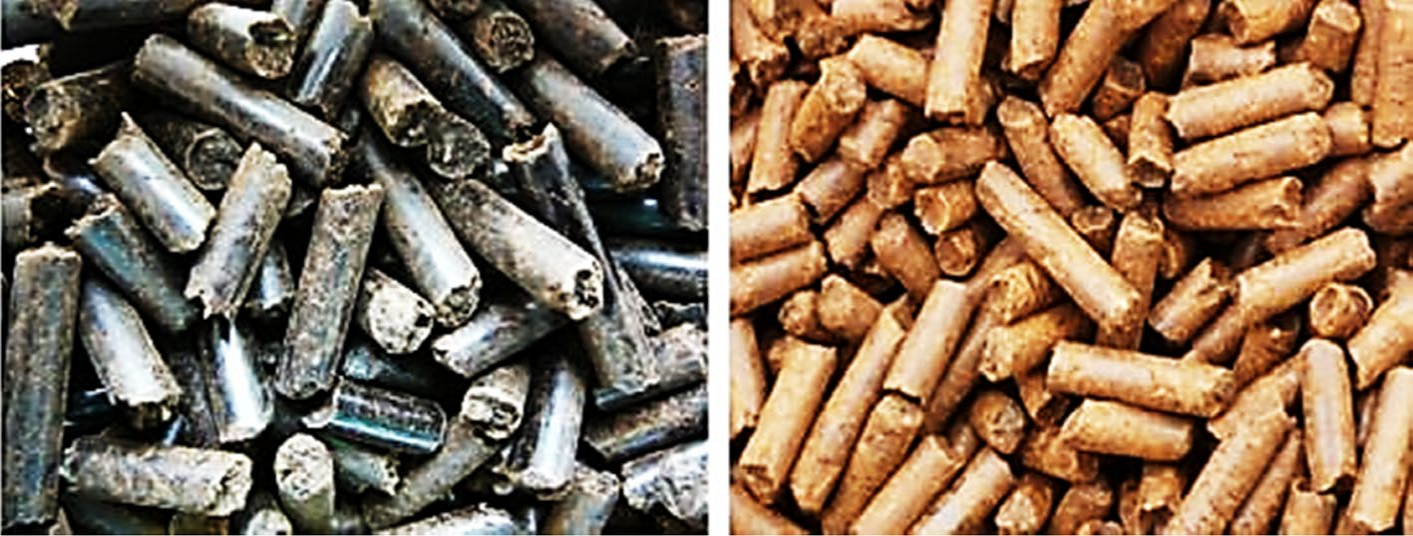
Samples of pellets (Graham et al. 2017)

**Fig. 4:**
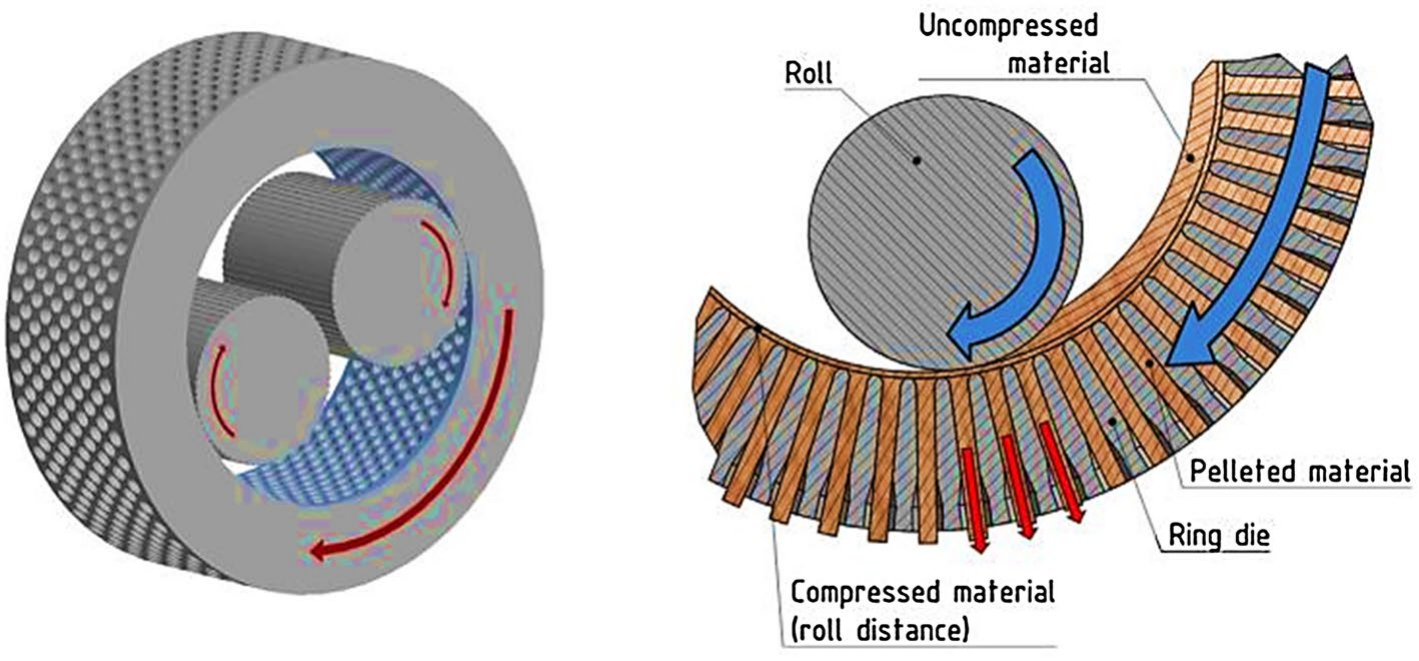
Schematics of ring die pellet press (Klinge et al. 2020)

Pelletizing has been adopted as a biomass waste management and processing technique and production of solid fuel for several applications. The product of pelletizing is referred to as pellet—a solid fuel that is characterized by high bulk and high energy densities. Some logistical characteristics such as storage, handling, and transportation are advantageous using pellets. Conversion of biomass into pellets considerably decreases dust generation, reducing agro-residues risks and negative effects during utilization, handling, and operations. Compared to briquetting process, the major difference is the dies. Pelletizing dies generally have smaller diameters (up to about 30 mm), and the machine has the dies arranged as boring holes in a thick steel disc ring. The roller of the die is used to press the feedstock into the holes. Ring and flat die are the two main types of pellet press (Stelte 2011; Djatkov et al. 2018; Bhattacharya and Salam 2014). The pellets are ejected hot from the dies, followed by cutting to lengths of about two times the diameter (Oladeji 2015). The flat type is made with a circular holed disk on which the rollers rotate, while ring types are made with a rotating holed ring on which the rollers press against the internal boundary. The capacity of the pellet press is independent of the density of the feedstock, which makes it different from piston or screw presses. Roller press with a cog-wheel and circular die is the best-standardized pellets machine (Oladeji 2015; Sugathapala and Chandak 2013). This machine was initially developed for animal feed production. It functions by extruding the pellets through a multiple holed die (Oladeji 2015; Sugathapala and Chandak 2013). Figure 3 shows samples of pellets, while Fig. 4 displays the schematics of the die pellet press.

Production of pellets with good physico-mechanical properties greatly depends on two essential parameters: process and feedstock parameters. Particle size distribution, moisture content, and homogeneous distribution of blend materials are essential feedstock parameters (Kirsten et al. 2016). The feedstock parameters significantly influence the properties of pellets. A Feedstock with a closely packed particle distribution is likely to produced pellets of high density. The production of pellets at optimum moisture content usually results in pellets of good characteristics. Though, the optimum moisture content differs for all feedstock. The feedstock moisture content dramatically influences the durability of the pellet. Also, feedstock particle size distribution significantly affects the physico-mechanical properties of pellets- bulk, green, and relaxed densities, compressive strength, impact and water resistance, and durability.

The essential process parameters include the die geometry, die and roller clearance, and capacity of the press (flow rate). The most important process parameters are compression pressure and temperature (Kirsten et al. 2016). The process parameters are interconnected; an increase of one parameter might lead to a decrease or increase of another parameter. For example, an increase in temperature would lead to a reduction in pelletizing pressure. Also, pelletizing pressure usually increases as feedstock particle size decreases. Die geometry, roller-die clearance also influence the characteristics of pellets. The die diameter significantly affects the density and durability of produced pellets. Larger die diameters haves pellets of high density with good durability properties, though the effect of die length on pellet properties was reported to be insignificant (Bhattacharya and Salam 2014; Kirsten et al. 2016).

Production of pellets from biomass such as agro-residues requires an understanding of the biomass bonding mechanism. Agro-residues are usually retained together by interlocking bonds. Thus, an appropriate particle size distribution is required to close the holes and gaps between particles during pellets production. Similar to briquetting process, the addition of a binding agent or adhesive could enhance biomass pellets’ bonding and strength properties. For woody biomass, particles are retained together by solid bridges through softening of lignin and inter-diffusion of adjacent particles. Furthermore, the formation of bridges can occur with natural binders such as proteins, starch, and lignin at particular process temperatures and water contents. Hydrogen bonding and Van der Waals forces are also significant in wood pellet formation (Kirsten et al. 2016; Lestari et al. 2017). Most of the time, woody biomass is the major feedstock used to produce pellets. Yet, there are areas where wood is not available or inadequate to meet the prevailing market demands of biomass fuels. This is predominant in intensive agriculture, where agricultural residues are available in large quantities and at lower costs than wood (Djatkov et al. 2018).

It is worthy of note that any feedstock considered for pellets production must possess sufficient energy content. Feedstock energy contents are measured in terms of energy density- energy per unit weight or volume. The energy density per unit volume of feedstock is significant given the volume of feedstock needed to be utilized in the energy conversion process. Feedstock with higher energy density requires less volume of feedstock to generate pellets of a given amount of energy content (Zych 2008).

## Characterization of feedstock and densification products

For biomass to be rationally and efficiently utilized as solid fuel, it must be characterized in order to determine its fuel properties (Mohammed and Olugbade 2015). Depending on the measured properties, characteristics of biomass feedstock and densified biomass products can be categorized into mechanical, combustion, thermal, chemical, and physical characteristics (Oladeji and Enweremadu 2012). Fine particulate matters and particle sizes and shapes are also essential characteristics that must be assessed to determine the appropriateness of any raw material for briquette/pellet production (Asamoah et al. 2016; Nataša et al. 2017). Table 2 displays the essential feedstock and solid fuel characteristics for sustainable energy production.

**Table 2:** Essential feedstock and solid fuel characteristic

Classification	Properties	Descriptions	Methods/equations/equipment	References
Physical	Bulk density^a^	Density of feedstock. It influences the economics of storage collection and transportation	Ratio of measured mass (using any analytical balance) and calculated volume of feedstock	Lestari et al. (2017), Goulart and Maia (2013), Zhang et al. (2012)
	Green density^b^	The density of the solid fuel immediately after ejection from the mold	Ratio of mass to volume; measuring the mass and calculating the volume of the briquettes/pellet	Oladeji et al. (2016), Lestari et al. (2017), Goulart and Maia (2013)
	Relaxed density^b^	The density of the solid fuel after drying. It is the density of the fuel when it had achieved a stable weight	Ratio of mass to volume of the briquettes/pellet	Oladeji et al. (2016), Lestari et al. (2017), Goulart and Maia (2013)
	Water-resistance/porosity index (PI)^b^	The quantity of water the fuel will be able to absorb when exposed to a humid environment. Porosity affects the heat and mass transfer, airflow velocity, which in turn influences the heat conductivity, conversion efficiency, emissions and burning rate	It is calculated using the following expression: ${\mathrm{PI}}= \frac{\mathrm{MW}}{\mathrm{MF}}\times 100$ (1) where ${\mathrm{MW}}$ is the mass of water absorbed while ${\mathrm{MF}}$ is the mass of fuel sample	Tuates et al. (2016b), Oyelaran and Tudunwada (2015), Zhang et al. (2012)
	Particle distribution^a^	The particle size distribution influence the heat, diffusion, flowability, bonding and reaction rate	It is determined by performing sieve analysis	Tuates et al. (2016b), Zhang et al. (2012)
Mechanical	Compressive strength^b^	Measure the resistance of the solid fuel to squeezing and pressing forces	It can be determined using universal testing machine (UTM) in accordance with established standards	Paper and Luttrell (2012)
	Durability/shatter index^b^	Measure the degree of fuel breakage and shattering tendency under sudden forces	It can be determined by performing a drop test. Fuel with known weight and dimensions would be dropped on the concrete floor from a height of 1 m Calculate the shatter index (SI) after 4 drops $\% {\text {weight loss}}= \frac{{w}_{1}-{w}_{2}}{{w}_{1}}$ (2) ${\mathrm{SI}}=100-\% {\text{weight loss}}$ ${w}_{1}$ and ${w}_{2}$ are the weight of the fuel before and after shattering, respectively	Paper and Luttrell (2012)
	Impact/attrition resistance^b^	Measure the resistance of the solid fuel to impact and grinding forces	Tumbler could be employed to determine attrition resistance. A fuel of known weight is placed in a tumbler rotating at about 12 revolutions per minute for about 4 min. After the tumbling process, fuels are taken out and weighed. The expression used for the shatter resistance will be adopted to calculate the abrasive resistance	Paper and Luttrell (2012)
Combustion/thermal	Proximate analysis^a^	This analysis will reveal the feedstock moisture (MC), volatile matter (VM), ash (AC), and fixed carbon (FC) contents	The MC, VM, AC, FC can be determined following the procedures of ASTM E1871-82 (2006), E872-82 (2006) E1755-01 (2007) and E1756-08 (2008), respectively	Ikelle et al. (2014), Ajimotokan et al. (2019b), Chou et al. (2009), Young and Khennas (2003), Ghaffar et al. (2015), Shuma and Madyira (2019)
	Thermogravimetric analyses (TGA)^a^	Provide information on the thermal breakdown profile of feedstock. It measures the fuel percentage weight loss as a function of temperature and presents a peculiar shape as the resulting thermogram for fuel materials	Determine using thermogravimetric analyzer	Raj, et al. (2015), Anukam et al. (2017)
	Calorific value^a^	Reveals the feedstock energy potential	It is determined using bomb calorimeter or calculated from the results of proximate and ultimate analyses	Ikelle et al. (2014), Ghaffar et al. (2015), Shuma and Madyira (2019), Djatkov et al. (2018)
	Energy density/thermal efficiency^c^	Describe the amount of energy stored per unit volume. Thermal efficiency is the percentage of fuel energy available for power generation	It is usually measured by performing a water boiling test	Odusote and Muraina (2017)
	Ignition time^c^	Ignition time is the average time taken to achieve steady glowing fire while burning the fuel	Fuel ignition time is determined by burning a known quantity of the fuel in a charcoal stove	Oyelaran and Tudunwada (2015), Odusote and Muraina (2017)
	Combustion rate (CR)^c^	It is the time taken to burn a known mass of fuel completely	${\mathrm{CR}}= \frac{\text{Total mass of burnt sample}}{\text{burning time}}$(3)	Oyelaran and Tudunwada 2015, Odusote and Muraina (2017)
Chemical	Ultimate analysis^a^	Reveal the contents of hydrogen, nitrogen, sulfur, chlorine, oxygen, and carbon in the feedstock	Hydrogen, nitrogen, and carbon may be determined using an elemental analyzer, while sulfur may be determined using an atomic emission spectrometer	Thulu et al. (2016), Lestari et al. (2017), Gado et al. (2013)
	Chemical bonds and constituents and crystalline nature of feedstock^a^	Estimate the quality and quantity of the chemical constituents and crystalline nature of feedstock used for fuel production. Identification of the chemical bonds in the molecule and generate an infrared retention range of the compounds	These can be determine using Fourier transform spectroscopy (FTIR)	Onukak et al. (2017), Raj, et al. (2015), Anukam et al. (2017)
	Analysis of surface morphology^b^	It is used in portraying and distinguishing minerals and material formed together with surface components. SEM is used for viewing the surface morphology solid fuel to establish the suitability of fuel for a given application	Scanning electron microscope (SEM)	Promdee et al. (2017), Onukak et al. (2017)
	Elemental composition^a^	Used for quantitative and qualitative determination of elemental composition feedstock	X-ray fluorescence	Promdee et al. (2017)

^a^Characteristic of biomass feedstock

^b^Characteristic of densification product

^c^Characteristic of both biomass feedstock and densification product

## Advantages, disadvantages, and application of densification and its products

Densification and its products have numerous advantages and applications both in domestic and industrial settings. However, there few disadvantages associated with their utilization. Some of the advantages, disadvantages and application of densification and densification products are presented in Table 3 (Pimchuai et al. 2010; Tumuluru et al. 2011; Mopoung and Udeye 2017; Oladeji 2015; Thulu et al. 2016; Alhassan and Olaoye 2015; Grover and Mishra 1996a, b; Djatkov et al. 2018; Ahmed et al. 2008; Kaliyan and Morey 2009; Ilochi 2010; Deshannavar et al. 2018; Adu-gyam et al. 2019; Yusuf et al. 2021; Sakai et al. 2020; Mu et al. 2020; Lu et al. 2021; Singh et al. 2021; Shigehisa et al. 2014; Wang et al. 2014; Yank et al. 2016; Lubwama and Yiga 2017). Other treatments such as thermal treatment (torrefaction, carbonization, pyrolysis, and gasification) and biological treatment (anaerobic digestion) are also utilized for different applications. Products of carbonization and torrefaction can be used for household cooking and heating. Further treatment of these products via densification can raise their energy density. Products of gasification can be utilized in boiler and power plants for heat and electricity generation. Thermal treatment can also be utilized as a waste management technique for the control of greenhouse emissions. Other treatments such as pyrolysis and gasification can be employed to generate liquid and gaseous fuels, which can be utilized in automobile industries to power internal combustion and gas engines. Liquid and gaseous fuels can be utilized in household cooking and heating stoves. Products of anaerobic digestion are majorly gases that are rich in methane. The gas can be utilized through combustion for heat and electricity generation.

**Table 3:** Advantages, disadvantages, and applications of densification and densification products

Advantages	Disadvantages	Applications
Smaller storage space requirement and convenience to use Briquetting and pelletizing offer an inexpensive form of energy, creates eco-friendly environment, employment, and business opportunities Composition pellets/briquettes fuels are homogeneous Fuel pellets allow automation of stoking, thus improve the comfort for the user Briquetting/pelletizing manufacture fuel with high mechanical strength, uniformity, and heating value than its feedstock The use of briquettes/pellets in remote areas can reduce the time spent on gathering firewood, and this will reduce the risk associated with collecting firewood and saving the time for other profitable activities Briquetting and pelletizing preserve wood and preventing the destruction of forests	They are not applicable for the production of liquid fuel Additional energy input (essentially for drying, pressing, and grinding) increases the cost of production	Household cooking and heating using domestic stoves As a feedstock for the manufacture of iron and steel through direct reduction route using a tunnel and rotary kilns Co-fire in boiler, power plant for steam and energy generation Commercial waste management techniques and control of greenhouse emissions Food processing, dyeing, bleaching, and textile industries Generation of heat in the manufacture of clay products; Employed for generation of gas through Gasification processes Distilleries, water cleaning applications, restaurants, bakeries, and canteens Utilized for tobacco curing, oil milling, and tea drying Nutrient fertilizer and land restoration

The utilization of densification products can be maximized by upgrading their characteristics via thermal treatment, for example, carbonization and torrefaction. Carbonization or torrefaction of densification products improves their thermal, hydrophobicity, and combustion properties.

## Recent research efforts

This section presents the recent research efforts on biomass densification. The overview focused on articles that present results on factors that affect solid biomass fuels’ physical, mechanical, and combustion properties.

### Methods

An overview of recent literature was carried out by adopting the method used by Thürer et al. (2018). Only articles that present recent results on biomass (agro-residues) densification were searched and selected. As a result of large reporting and accuracy, reviewed articles were sourced from the ScienceDirect database to obtain high-quality articles. Selected articles were limited to peer-reviewed articles. ScienceDirect database was searched using the following search terms: densification; briquetting; briquette; pelleting; pellet; binder; additive; greenhouse gas emission; feedstock pretreatment; physical properties; mechanical properties; thermal properties; chemical properties and combustion properties. The keyword ‘biomass’ was utilized to bias the search from the database. To limit search results to a controllable article, searched results were restricted based on the article title and year of publication (from 2019 to 2021). However, very few articles directly related to the area of interest were considered in 2017 and 2018. Carefully chosen articles were analyzed based on the method of investigation, results, and conclusion.

### Overview of recent literature

Due to the renewed global interest in the development of alternative and environmentally friendly fuels from biomass feedstock to serve as a substitute to conventional fuels, great research efforts have been put into the investigation of factors that influence the physical, mechanical, chemical or compositional, combustion and thermal properties of solid fuels manufacture using biomass as feedstock (Ajimotokan et al. 2019c; Junga et al. 2021; Berdychowski et al. 2021; Thapa and Engelken 2019). These factors includes but not limited to moisture content (Berdychowski et al. 2021; Yang et al. 2021), particle size distribution (Olatunji et al. 2020; Matkowski et al. 2020a), process temperature (Berdychowski et al. 2021; Yang et al. 2021; Riva et al. 2019), present of additives(Song et al. 2019), blending of feedstock (Junga et al. 2021; Thapa and Engelken 2019), co-blending feedstock with coal, feedstock origin, compaction pressure (Ajimotokan et al. 2019c; Berdychowski et al. 2021; Yang et al. 2021; Song et al. 2021) and thermal pretreatment (Kang et al. 2020; Martín et al. 2020; Pawlak-kruczek et al. 2020). A detailed review of different factors that affect solid fuel quality can be found in Gilvari et al. (2019). Investigation was conducted utilizing feedstock from different origin such as Poland (Berdychowski et al. 2021), Colombia (Juan and Gonz 2020), India (Dhote et al. 2020; Rajput et al. 2020), Mississippi (Thapa and Engelken 2019), Korea (Park et al. 2020), Philippines (Navalta et al. 2020), Nigeria (Ajimotokan et al. 2019b), China (Xia et al. 2019) South Africa (Shuma and Madyira 2019), and Poland (Czeka et al. 2018) among other origins. Some of feedstock reported recently include cashew nutshell (Ifa et al. 2020; Chungcharoen and Srisang 2020), sugar cane bagasse (John et al. 2020; Setter et al. 2020), sawdust (Ajimotokan et al. 2019b; Yang et al. 2021; Afsal et al. 2020; Wang et al. 2020), rice husk and rice brain (John et al. 2020; Faverzani et al. 2020), palm kernel shell and oil palm fruit bunch (Cabrales et al. 2020; Osei et al. 2020), citrus peel (Faverzani et al. 2020), Sitka Spruce and olive pit (Trubetskaya et al. 2019), miscanthus, wheat, barley (Mitchell et al. 2020), areca nut (Chungcharoen and Srisang 2020), mushroom(Rafael et al. 2020) and biomass charcoal-based product (Ajimotokan et al. 2019b; Lubwama et al. 2020; Jelonek et al. 2020; Cong et al. 2020). Generally, government policy regarding renewable energy, greenhouse emission, and energy demand greatly determines the growth of solid biomass fuels in any region (Bajwa et al. 2018). The major global application of solid biomass fuels is for electricity generation and domestic and industrial heating (Bajwa et al. 2018). Studies were conducted to improve fuel characteristics on single feedstock as well as a blend of feedstock (Shuma and Madyira 2019; Martín et al. 2020; Rajput et al. 2020; Park et al. 2020; Navalta et al. 2020). For desirable coal-like performance, especially for industrial application, co-densification of biomass with coal or coke was studied (Ajimotokan et al. 2019b; Song et al. 2019). Densification of blends of biomass and co-blend with coal significantly improves solid fuel properties such as physical (density), mechanical (compressive strength), thermal (heating values), and combustion properties (proximate) properties (Navalta et al. 2020). A comprehensive review of the co-densification of biomass can be found in Kang et al. (2019).

Enhancing fuel properties such as physico-mechanical and combustion properties, binders (organic, inorganic, and compound), as well as some chemical substances, are included as an additive during the process of manufacturing solid fuels (Bajwa et al. 2018; Zhang et al. 2018). Different binders and a blend of binders have been reported in the literature to influence fuel properties (Zhai et al. 2018; Shuma and Madyira 2019). Examples of binders popularly utilized in the manufacture of biomass solid fuel are starch (Ajimotokan et al. 2019c; Navalta et al. 2020; Merry et al. 2018; Hu et al. 2019), molasses (Zhai et al. 2018; Wang et al. 2019; Barriocanal 2020), bio-tar (Cong et al. 2021), coal tar (Barriocanal 2020), xanthan and guar gums (Espuelas et al. 2020) thermoplastics (Song et al. 2021), pyrolysis oil (Riva et al. 2019), calcium carbonate (Matkowski et al. 2020b), glycerol contents (Martín et al. 2020; Juan and Gonz 2020; Xia et al. 2019; Azargohar et al. 2019), recovered polyvinyl alcohol (Rajput et al. 2020; Hu et al. 2019), waste cooking oil and waste lubricating oil (Rajput et al. 2020), paraffin (Xia et al. 2019; Barriocanal 2020), red clay and sodium humate (Song et al. 2019), cassava peel (Ajimotokan et al. 2019b), alkali lignin and L-proline (Azargohar et al. 2019), cow dung and cactus (Shuma and Madyira 2019) and calcium hydroxide (Merry et al. 2018). A comprehensive review of densification binders and densification mechanisms can be found in Zhang et al. (2018). Studies have been conducted using different percentages of binder and binder blends with other process parameters such as process temperature and compaction pressure to obtain fuel with optimum properties. Percentage rages of 5–10% (Espuelas et al. 2020), 2–10% (Matkowski et al. 2020b), 0–10% (Juan and Gonz 2020), 5% (Ajimotokan et al. 2019b), 1–10% (Xia et al. 2019), 10–20% (Wang et al. 2019), 4% (Merry et al. 2018) have been utilized. ISO standard specified the range of percentage (< 4 wt%) of binder that must be utilize for the development of solid fuel. Recently, a binder (PVA–EPC–peptides) was developed from animal protein and specified risk materials for solid production (Shui et al. 2020). At < 3 wt% binder, the developed binder displayed excellent binding property. Paraffin was also reported displayed good binding property at 4% addition in fuel production (Xia et al. 2019). Having investigated effect of using different binder for biomass densification purposes, Florentino-Madiedo et al. recommended bituminous binder especially when combined with lignin over molasses and paraffin binders due to its greater Gieseler fluidity, lesser emissions and better strength (Xia et al. 2019; Barriocanal 2020). For enhanced mechanical properties, l-proline and polyvinyl alcohol binder is highly recommended (Hu et al. 2019; Azargohar et al. 2019). Also, utilization of bio-tar, thermoplastic substantially enhance fuel physical and mechanical stability (Song et al. 2021; Cong et al. 2021). Addition of plastic up to 10% at 300 kN compacting force will produce fuel with optimum properties comparable to coal (Song et al. 2021). Particle size and shape as well as their distribution affect the bonding mechanism which in turn influence the solid fuel quality (Matkowski et al. 2020a). Evaluation was conducted on the effect of natural binding characteristics of feedstock on briquettes process parameters (Afra et al. 2021). It was discovered that nano-lignocellulose and nano-cellulose binders displayed better binding properties when compared with lignin binder.

The utilization of some additives has a negative impact on fuel properties. For example, the addition of a binder such as bio-tar was reported to increase greenhouse gas emissions during fuel combustion. However, the addition of 3 wt% of hydrated lime eliminates or weakens the greenhouse gas effects (Cong et al. 2021). In addition, acidified calcium oxide has been utilized as desulphurized, while the blend of molybdenum and calmogastrin has been reportedly used as a smoke suppressor (Song et al. 2019). The emission of ultrafine particulate matter during biomass fuel combustion poses a great environmental threat to people. But recently, phosphoric acid-modified kaolin was developed as a fuel additive to mitigate this effect with emission reduction capability and achieve higher ash fusion temperature and slagging tendency (Kri et al. 2018; Cheng et al. 2021; Gehrig et al. 2019). Little addition of kaolin (0.2 wt%) can reduce particulate emission. However, the emission capacity was reported to be proportional to acid concentration (Cheng et al. 2021; Gehrig et al. 2019).

Biomass densification can be carried out at room temperature (Espuelas et al. 2020). However, preheating feedstock before densification enhances physico-mechanical properties (Ajimotokan et al. 2019a; Ojolo et al. 2016). Thermal pretreatment was reported to improve fuel thermal and combustion properties (Xia et al. 2019; Cong et al. 2021; Sharma and Dubey 2020). Cong et al. reported that increasing densification temperature beyond 20 °C would negatively affect the mechanical fuel property (Cong et al. 2021). On the contrary, higher densification temperature was reported to give optimum performance (Junga et al. 2021; Berdychowski et al. 2021; Riva et al. 2019). According to the report of Navalta et al., mechanical densification does not have a significant impact on the combustion characteristics of solids fuel (Navalta et al. 2020). However, mechanical densification increases energy, compresses, and relaxes densities (Ajimotokan et al. 2019c). Treating solid fuel via thermal process is recommended for the improvement of combustion properties especially when the fuel is targeted towards industrial applications (Riva et al. 2019; Navalta et al. 2020; Xia et al. 2019; Bajwa et al. 2018). Combustion properties can be promoted by adding either citric acid, KNO_3_ or MnO_2_ to the feedstock (Song et al. 2019).

Yang et al. recommended a densification pressure greater than 38 MPa at 8–10% moisture content to optimum fuel durability (Yang et al. 2021). This recommendation was in line with the report of Berdychowski et al. (2021).

## Drawbacks and proposed possible solutions

This section presents the currently known challenges and drawbacks associated with biomass densification and proposed possible solutions.

### Drawbacks/challenges

Densification success is recorded mostly in the developed nations. Developing nations experience drawbacks due to poor management, inadequate equipment, and high investment costs. Potential increases in corrosion and fly ash are experienced with biomass pellets/briquettes—differences in the composition of biomass influence densification characteristics. Feedstock cultivation is affected by weather conditions. Biomass absorbed moisture when exposed to a humid environment—external energy required to dry feedstock with high MC and contributed to production cost. Transportation of feedstock from field to briquetting/pelletizing site is usually very difficult and labor-intensive. During transportation and processing, clouds of dust are generated, which have a negative impact on the operator’s/workers’ health. There is inefficient and ineffective utilization of densification products as a result of a lack of awareness. Combustion of biomass briquettes generates some emissions, though lesser than that of fossil fuel. Competition between food and feedstock, especially feedstock that is edible to humankind, is another challenge. Some government policy does not encourage the application of biomass briquettes/pellets in domestic and industrial settings.

### Possible solutions

Densification products should be kept in an air-tight bag or environment. Optimization of the densification process and preliminary characterization of feedstock will help to reduce the effect of biomass type and biomass composition on the densification products. Biomass thermal pretreatment techniques are recommended to improve the hydrophilic nature of biomass. Thermally treated biomass possesses good hydrophobic property. Solar energy can be utilized to dry fresh biomass. The densification site should be located in the field or close to the source of feedstock. Personal protective equipment should be used to minimize the effect of dust during operation and transportation. A sensitization program should be organized to inform people of the potential, effectiveness, and use of biomass densification products for different applications. This program is highly recommended in rural areas. Non-food feedstock should be utilized more than feedstock that also serves as food for humankind. Government should make a policy that encourages biomass briquettes/pellets for domestic and industrial applications. This will reduce the harmful emissions due to the use of fossil fuels and promote a green environment. Government should assist energy industries through credit facilities, procurement of expensive densification equipment, and tax relief.

## Recommendation for further research

The techno-economic analysis on the manufacture of fuel briquettes and briquettes from a blend of feedstock should be carried out. Biomass thermal pretreatment before densification and thermal treatment after densification should be studied, and results should be compared using the same feedstock. Further study is required to establish qualitative, quantitative, and rapid characterization methods for densified products. Optimization of the physical, mechanical, and chemical treatments desirable for different feedstock suggested for further study. There is little or no commercial manufacture of biomass pellets/briquettes in many developing countries. R&D should seek to move from laboratory to commercial scale in these areas. Strong business plan and implementation skills should be developed to achieve greater commercial success. An in-depth fuel characterization using a blend of agro-residues, especially blends of corncob, rice husk, and groundnut shell, is recommended for further studies.

## Conclusion

An overview of densification technologies (pelletizing, briquetting) as efficient and convenient methods for providing energy was presented in this article. Densification of biomass has moderate operating costs. The advantages, disadvantages, and applications of densification and its products were enumerated in the article. Essential fuel characteristics (physical, mechanical, thermal, composition and combustion) were discussed, and known drawbacks and possible solutions. Solid fuel production via densification could provide substantial and important socio-economic and environmental benefits.

## Data Availability

The data that support the findings of this study are available within the article.
